# A new expansion material used for roof-contacted filling based on smelting slag

**DOI:** 10.1038/s41598-021-81891-4

**Published:** 2021-01-28

**Authors:** Hua Na, Guocheng Lv, Lijuan Wang, Libing Liao, Dan Zhang, Lijie Guo, Wenchen Li

**Affiliations:** 1 School of Materials Science and Technology, Beijing Key Laboratory of Materials Utilization of Nonmetallic Minerals and Solid Wastes, China University of Geosciences, Beijing, 100083 China; 2 BGRIMM Technology Group, Beijing, 100160 China

**Keywords:** Materials science, Structural materials, Mechanical properties

## Abstract

The improper handling of smelting slag will seriously pollute the environment, and the unfilled roof of the goaf of the mine will threaten the safety of the mine. Expansion materials have attracted more and more attention because of their excellent properties. In this paper, copper-nickel smelting slag that has some active ingredients of gelling is used instead of traditional aggregate and some part of cement in order to reduce its pollution to the environment and its costs. For safety reasons, hydrogen peroxide was chosen as the foaming agent. Sodium silicate and hexadecyl trimethyl ammonium bromide (CTAB) are used as additives. Our results showed that after 28 days of curing, the material has better mechanical properties and the early compressive strength of the material was enhanced by sodium silicate. The efficiency of foaming was improved by CTAB. It also proves that copper–nickel smelting slag can be used in expansion material. At the same time, the utilization rate of the copper–nickel smelting slag of this formula can reach 70%, reduce its pollution to the environment.

## Introduction

Mineral resources are one of the most critical parts of the development of society. Filling mining technology is a mining technology that uses filling materials to fill underground goaf. There are many kinds of filling materials, such as barren rocks, slags, tailings, cementitious material and cementitious material prepared with cement and the solid waste mentioned above in the land mines^1–8^. Moreover, the Sea sand and coral sand are also used in the Marine concrete engineering, especially in the reef construction in the open sea^9^. Most solid wastes in mining areas still have many harmful substances even after smelting. When piled up in the open air, under the erosion effect of the natural environment, the harmful substances in these wastes will be dissolved and leachable, which will spread to the surrounding areas and infiltrate into the underground water system at the same time, causing incalculable damage to the surrounding environment and organisms. Some of the solid waste in mining area which has particular cementing activity and can partially substitute cement for cementing, so solid waste in mining area can be used as a component to prepare to fill cementing materials during filling mining operations. Not only can it improve the utilization rate of solid waste, reduce the environmental pollution caused by its accumulation, but also reduce the cost of cementitious materials. Filling cementitious materials have become one of the research hotspots in the mining industry.

At present, the way to transport the filling material in mining areas is through pipelines. Therefore, too much water will be added in the filling material for adequate liquidity in order to make it be flown smoothly through the pipelines into the underground goaf. After the filling material gets into the underground goaf, the material is unable to reach the top because of the solid particle sedimentation. Furthermore, the artesian slope, which causes the filling around the pipeline not to be topped, will form during the descent of the filling material. There are also other factors that affect roof-contacted filling, such as the irregular shape of roof, atypical mining technology and some human factors. A roof-contacted filling is an important factor affecting stope filling effect and directly affects the stress distribution and stability of surrounding rock. It could cause underground stress imbalance, then rock failure would happen. The safety of mine workers is threatened. Roof-contacted filling has been one of the series of security issue in mine lot.

People gradually notice expansion materials because of their useful properties, including lightweight, high intensity, heat insulation, sound insulation, energy absorption, humidity adjustment, high fluidity and so on^10–13^. It has been studied and applied in many fields. Flexible foams are used to design for a football helmet’s top pad^12^. A phase transition/graphite foam composite material has been prepared and the heat storage capacity, stability and mechanical properties of this material were analyzed^14^. The microwave absorption performance of SiC/C foam material with that of SiC/C bulk material was compared and proved that the microwave absorption performance of SiC/C foam material is stronger than SiC/C bulk material because of the foam structure^15^. An inorganic thermal insulation material was prepared with perlite tailing plus sodium silicate, which showed suitable physical property: lower thermal conductivity and lower density^16^. However, the research and application of expansion material applied to mine filling roof are relatively few.

Expansion materials are usually composed of cementitious material, aggregates, additives, foaming agents and water. Traditional aggregates could provide strength to the material. In order to reduce environmental impact of solid waste in mining areas, some or all of the aggregate is replaced by solid waste. The composition of solid waste is complex, and most of the solid waste contains components with gelation activity. Therefore, in addition to being used as aggregate, it can also replace part of cement^17–19^. Additives include water reducers, thickeners, surfactants, chemical activator, foam stabilizers and fibers. Additives can increase the stability of the expanded material system, improve the fluidity of the slurry, and adjust the uniformity of the pore structure and the speed of foaming at the same time. Although the addition of additives is not large, it is closely related to whether the foaming can proceed smoothly and meet the design requirements. Surfactants can reduce the density of expanded materials increase their porosity, lower the surface tension of the liquid and make the area of the gas–liquid interface larger^20,21^. Reducing the surface tension of the liquid by surfactants can significantly improve the foaming ability of the aqueous solution^22,23^. The liquid film adsorbed by surfactant molecules has excellent elasticity, which makes the bubble self-repairing ability stronger as well as the bubble more stable^24–26^. The chemical activator provide a different chemical environment, such as alkaline activators can provide an alkaline environment, which can promote the hydration reaction of cement and improve the mechanical properties of the material^27–29^. However, it is also necessary to control the optimal ratio of additives according to the mechanical properties of the material at the same time.

The most common route to produce foams is by the incorporation of a foaming agent into the slurry^30–32^. There are two different methods have been adopted to swell the material. One is the Physical foaming method. The physical foaming agent is processed by machine, and then mixed with the slurry to achieve the purpose of foaming^33^. Such as plant foaming agent, surfactant foaming agent and so on. Modified sodium alcohol ether sulphate and plant fiber are used to prepare expansion materials for energy absorption with good physical property, better porosity and thermal conductivity^34,35^. The Phase transition/graphite foam prepared with polyurethane also has batter properties^14^. In general, the void sizes in foamed concrete produced by physically foaming are better^36^. The other is the chemical foaming method. The chemical foaming agent produces gas in the slurry making the material expansion. Such as hydrogen peroxide, fine metallic powders^37,38^. The mechanical property of material that was foamed by hydrogen peroxide is better^39^, and the foam concretes which was prepared with aluminum powder have more uniform pore structure^40^. The better mechanical property of material could be given by hydrogen peroxide. The theory of aluminum powder foaming is that the aluminum powder reacts with the water and hydroxyl radical to form hydrogen, which make the material expand. Hydrogen peroxide also causes the material to expand when it breaks down on its own^41^. Compared with aluminum powder, the oxygen produced by the breakdown of hydrogen peroxide is safer than the hydrogen produced by adding aluminum powder. Research has shown that that the compressive strength and density of the fly ash-based expansion material which was prepared with hydrogen peroxide are higher than this material which was prepared with aluminum powder^41^. So hydrogen peroxide is a safer and more efficient foaming agent than aluminum powder.

In this study, copper-nickel smelting slag was used instead of traditional aggregate and some part of cement. Hydrogen peroxide was used instead of aluminum powder as foaming agent. Hexadecyl trimethyl ammonium bromide (CTAB) was used as surfactant and sodium silicate (SS) was added to provide an alkaline environment. 42.5 cement was used as the cementing material to prepare cementitious material, which was used for roof-contacted filling had good physical and mechanical properties. In this paper, the method of controlling a single variable is used to determine the best ratio of cementitious material, aggregate and additives from the aspects of compressive strength (CS), density, porosity and composition, then the test results of each ratio are discussed.

## Materials and methods

### Materials

Copper-nickel smelting slag is from Xinjiang Kalatongke Mining Co., LTD. (Altay, China), which was supplied by Beijing General Research Institute of Mining & Metallurgy. Because the slag size is large, it will be crushed by ball mill. 425 cement was purchased from China Building Materials Academy. Sodium silicate was procured from Beijing Hongxing Guangsha Chemical Building Materials Co. LTD. CTAB was purchased by Tianjin Guangfu Fine Chemical Research Institute.

### Method

In this study, 425 cement is used as a cementing material, copper-nickel smelting slag is used as aggregate. CTAB and sodium silicate are additives. First, the slag was grinded in a horizontal ball mill for 6 h at 1000 r/min, then mixed the cement with smelting slag in the sand-cement ratios(S/C) of 4:1, 7:3, 3:2, 2:3, 3:7 and 1:4, CTAB and water were added and stirred for 10 min. After that added 5% hydrogen peroxide and stirred for 1 min. Poured the slurry into a 50 mm × 50 mm × 25 mm silicone mold. Samples with different ratios of sand to ash are denoted as A-1 to A-6. After that kept it at room temperature for 24 h. Finally placed it in a 99% humidity curing box for 3, 7 and 28 days, then took it out. The compressive strength was measured after drying for 24 h at room temperature. After that the best S/C which compressive strength of sample is higher than 1 MPa after 28 days cured was selected. Different sodium silicate was added. Samples with different doses of sodium silicate are denoted as B-1 to B-5. After curing for 3 d, 7 d, 28 d, the compressive strength test was carried out. After selecting the optimal addition amount, different doses of CTAB were added. Samples with different doses of CTAB are denoted as C-1 to C-5. The compressive strength test was performed after 3, 7 and 28 days. Then chose the best ratio of cement, aggregate and admixture.

### Characterization

The primary crystal phase of a sample was analyzed by X-ray Diffraction (XRD, Rigaku, D/max 2500 V). The microstructure of samples was observed via Nova NanoSEM450 field emission scanning electron microscope (FEI Company, America).

The samples were cured in standard constant temperature and humidity curing box (YH-40B). The compressive strength of the samples was tested by tension machine sensor (304C, Shenzhen Wance Testing Machine CO., LTD., China). Three samples were tested in each group. The density of the samples was calculated by the mass and dimensions method. The dimensions of all samples processed into regular shapes were measured by Vernier calipers. Three samples were measured in each group. The porosity of the samples was measured by volume difference. The sample of known volume is ground into powder then pressed into a disc by power compressing machine (769YB-24B, Tianjin Keqi High & New Technology Corporation, China). The volume of the disc is measured with Vernier caliper. The volume of the measured disc is divided by the original volume to obtain the porosity.

## Results and discussions

### Raw material analysis

**Figure 1:**
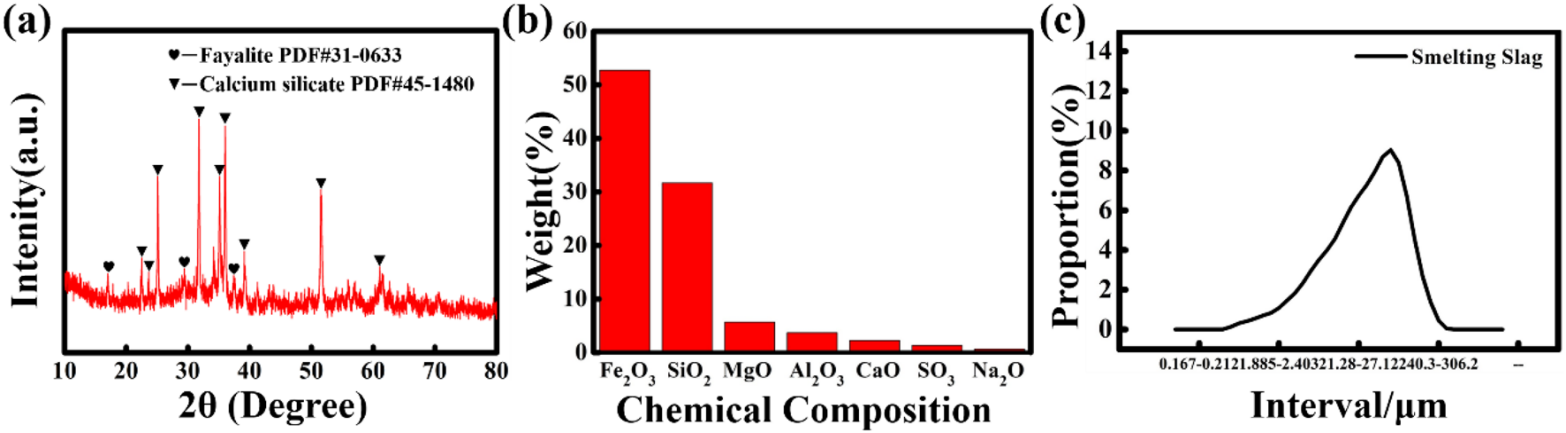
Analysis of smelting slag characteristics. (**a**) XRD pattern; (**b**) XRF analysis; (**c**) the particle sizes range.

The phase was analyzed with X-ray diffraction (XRD) pattern. The results of copper-nickel smelting slag after grinding are shown in Fig. 1a. As can be seen from the figure, the smelting slag is mainly composed of fayalite and a small amount of calcium silicate hydrate. The X-ray fluorescence (XRF) analysis of the copper-nickel smelting slag are shown in Fig. 1b. From this figure, it is shown that the primary ingredients are Fe_2_O_3_, SiO_2_, MgO, Al_2_O_3_, CaO, SO_3_ and some heavy metal oxide like CuO, NiO, TiO_2_, Co_3_O_4_. Figure 1c shows the morphology of the copper-nickel smelting slag which reveals that the copper-nickel smelting slag particles are irregular in shape with the particle sizes range from 20 to 30 μm.

P.O 42.5 cement is used for cementing agent. The main mineral composition of Portland cement is calcium silicate, dicalcium silicate, tricalcium aluminate and calcium aluminosilicate. This is consistent with the mineral composition of cement clinker.

### The effects of different sand cement ratio on the performance of the expansion materials

**Figure 2:**
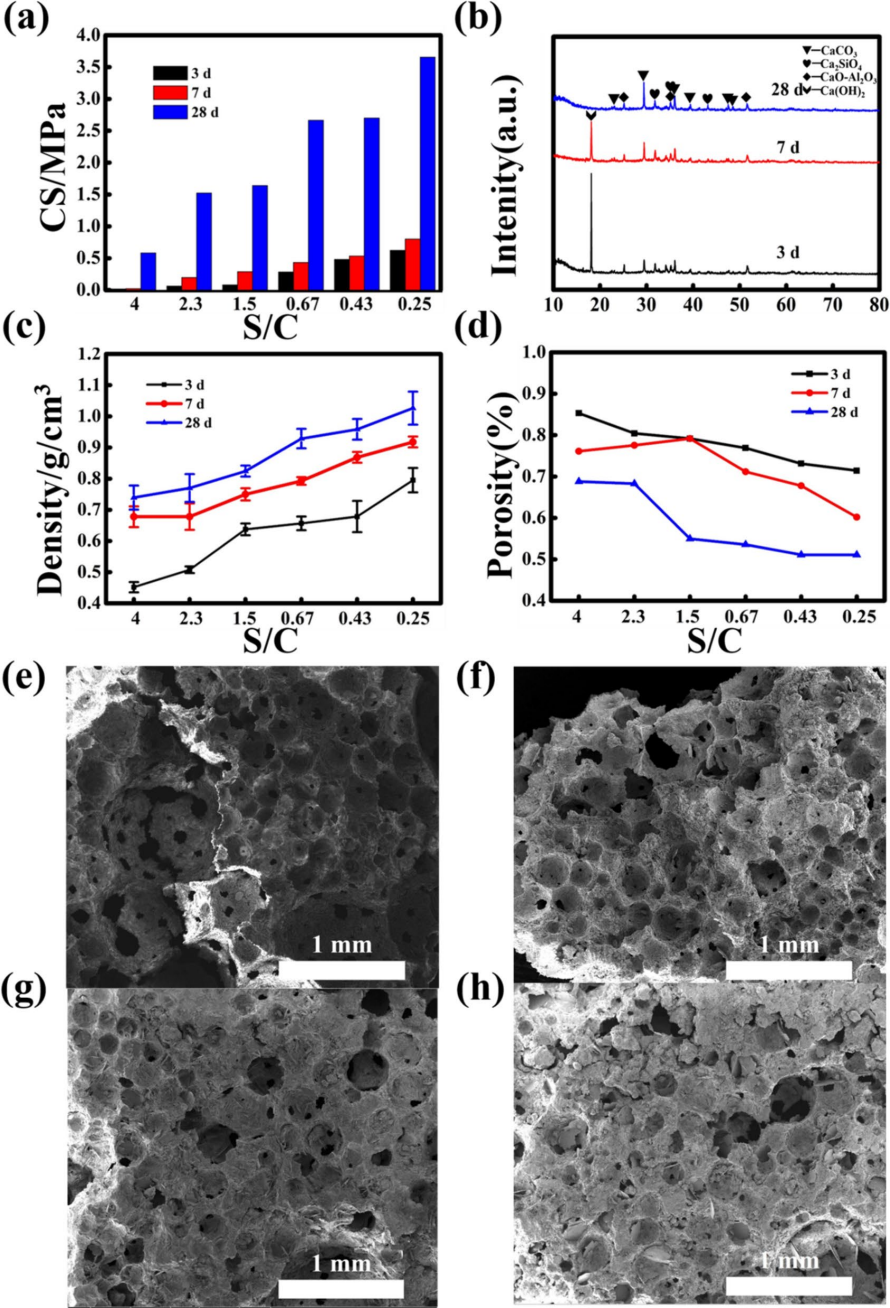
Properties and microstructure of expansive materials with different sand-cement ratios. (**a**) compressive strength, (**b**) XRD pattern of A-2 cured for 3 d, 7 d, 28 d, (**c**) density, (**d**) porosity, (**e**,**f**,**g**,**h**) SEM images of A-1, A-2, A-6-A-7 cured for 28 days.

In order to find the best sand-cement ratio, the experimental program is shown in Table 1. Choose the sand-to-cement ratio as 4:1, 7:3, 3:2, 2:3, 3:7 and 1:4. After curing for 3 days, 7 days, and 28 days, the compressive strength was tested. The test result is shown in Fig. 2a. As the proportion of cement increases, the compressive strength of the test block also increases. When the sand-to-cement ratio is lower than 2:3, the compressive strength of the test block is higher. After 28 days of curing, the compressive strength of the test block with a sand-to-cement ratio of 3:7 can reach 1.52 MPa. The test block with a sand-to-cement ratio of 1:4 has the highest compressive strength, reaching 3.65 MPa. This is because cement is a good cementitious material^42^, after hydration reaction can have better compressive strength. The higher the amount of cement added, the more cement hydration products and the greater the compressive strength of the sample (Figure S1). The compressive strength of the samples of group A-2 has reached 1 MPa. In order to increase the utilization rate of smelting slag as much as possible, the subsequent experiments select the sand-to-ash ratio of 3:7 for an experiment.

**Table 1:** Experimental scheme for the influence of sand-cement ratio, Sodium Silicate and CTAB to the material.

Sample number	S/C	SS, wt%	CTAB, wt%	H_2_O_2_, wt%
A-1	4	0	0.26	4
A-2	2.3	0	0.30	5
A-3	1.5	0	0.30	5
A-4	0.67	0	0.32	6
A-5	0.43	0	0.32	6
A-6	0.25	0	0.34	7
B-1	2.3	1	0.30	5
B-2	2.3	2	0.30	5
B-3	2.3	3	0.30	5
B-4	2.3	4	0.30	5
B-5	2.3	5	0.30	5
C-1	2.3	2	0.15	5
C-2	2.3	2	0.30	5
C-3	2.3	2	0.45	5
C-4	2.3	2	0.60	5
C-5	2.3	2	0.75	5

Figure 2b shows the XRD patterns of group A-2 after curing for 3 days, 7 days, and 28 days. It can be seen from the figure that with the increase of the curing days, the higher the hydration products of cement, which proves that the curing time determines the compressive strength of the material. A strong Ca(OH)_2_ peak can be seen in the figure of curing for 3 days and 7 days. The main components of cement, dicalcium silicate and tricalcium silicate, can react with water to generate Ca(OH)_2_. When the curing age reaches 28 days, the Ca(OH)_2_ phase disappears. The reason is that Ca(OH)_2_, the active SiO_2_ and Al_2_O_3_ in the smelting slag reflect the formation of hydrated calcium silicate and hydrated calcium aluminate^43^, These two substances play an important role in maintaining the early strength of the test block.

Figure 2c,d shows the density and porosity of the sample. It can be seen from Fig. 5 that the sample density is negatively correlated with porosity and positively correlated with cement proportion. We can know from Fig. 2d that the porosity of the specimen cured for 3 days is not much different from that of the specimen cured for 7 days, but the porosity of the specimen cured for 28 days has a significant decrease. The reason is that after the curing age of 28 days, the cement in the test block has been fully reflected with water to generate a large number of hydration products, which leads to the decrease of its porosity. This is consistent with the results observed from the SEM image (Figure S2).

Figure 2e,f are scanning electron microscope image of some samples. When the cement proportion is relatively low, the pore wall of the sample is smooth and the pore structure is relatively uniform as well as dense. When the cement proportion is high, the pore structure of the sample is uneven and the distribution is loose. At the same time, there are a lot of hydration products in the pores. This results in a decrease in sample porosity. The pore structure contains many voids, which proves that during the foaming process of the sample, when the gas gathers to a specific size, it will break through the sample and form new pores above it. When the slurry solidifies, the pore structure is left inside the sample, forming an expanded material.

### The effects of different doses of sodium silicate on the performance of the expansion materials

**Figure 3:**
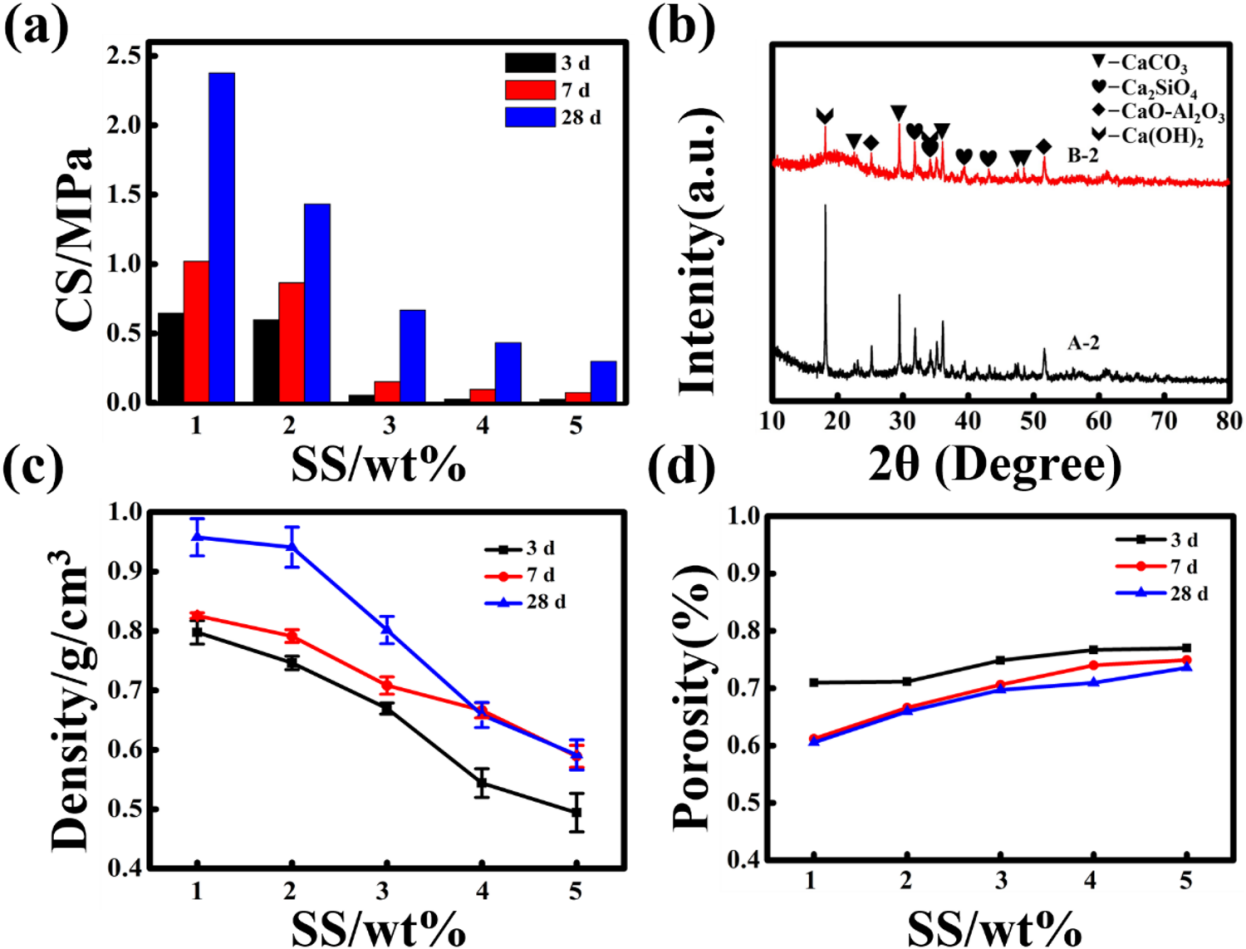
Properties of expansive materials with different doses of sodium silicate. (**a**) compressive strength, (**b**) XRD pattern of group A-2 and B-2 samples after cured for 7 d, (**c**) density, (**d**) porosity.

In the above experiments, the compressive strength of the samples of group A-2 has reached 1 MPa. In order to explore the effect of the amount of sodium silicate added on the properties of samples, the experimental scheme is shown in Table 1. Add 1%, 2%, 3%, 4%, 5% sodium silicate, respectively. After curing for 3 days, 7 days, and 28 days, the compressive strength was tested. The experimental results are shown in Fig. 3a. It can be seen from the figure that the compressive strength of the sample decreases with the addition of sodium silicate. The addition amount of 2% and below 2% of sodium silicate can reach above 1 MPa. Compared the compressive strength of the sample with sodium silicate with the sample without sodium silicate, it can be seen that the strength of the former is significantly higher than the latter in the initial stage of hydration. This is because the hydrolysis of sodium silicate to generate a large amount of silicate ions and hydroxide ions. The hydroxide ions react with the active SiO_2_ in the smelting slag to form a hydrated calcium silicate gel with high calcium to silicon ratio. The higher the Ca–Si ratio, the greater the strength provided by the product. The hydrated silicon calcium acid gel is the main substance that provides the strength of cement. In addition, there are always a large number of free active silicate ions in the material and free calcium ions in the concrete that have not reacted to form calcium silicate hydrate (C–S–H), so that the calcium ions in the concrete can further participate in the reaction and greatly improve the strength and hardness of concrete. This improves the early strength of the sample. A large number of studies have shown that the strength of cement depends on the early strength. The addition of sodium silicate increased the ratio of calcium to silicon. So the addition of a small amount of sodium silicate can improve the material performance^44–47^. Figure 3b shows the XRD patterns of samples A-2 and B-2 cured for 7 days. It can be seen from the figure that after 7 days of curing, the B-2 sample contains more calcium silicate, which is consistent with the previous analysis results. When the amount of sodium silicate is too high, the hydration reaction of cement will be affected. When the amount of sodium silicate added reaches 5% of the total dry weight, there is almost no Ca(OH)_2_ in the samples at the initial stage of curing. It is proved that excessive sodium silicate will inhibit the hydration reaction of cement (Figure S3).

With the increase in the amount of sodium silicate added, the decomposition rate of hydrogen peroxide slows down, which in turn leads to an increase in porosity. This resulted in a decrease in the compressive strength of the sample. As shown in Figure S4, as the amount of sodium silicate increases, the pore volume of the sample increases. The density and porosity of the sample are shown in Fig. 3c,d. The density and porosity of the sample decreased with the increase of sodium silicate. However, the porosity of the samples did not change so much. Although the compressive strength of the sample with 1% sodium silicate addition also reached the standard (1 MPa), it can be seen from Fig. 3d that the porosity of the sample with 2% sodium silicate addition is better than the sample with 1% sodium silicate addition. Therefore, according to analyzing the compressive strength and porosity of the samples, the addition amount of 2% sodium silicate is selected for subsequent experiments.

### The effects of different doses of CTAB on the performance of the expansion materials

**Figure 4:**
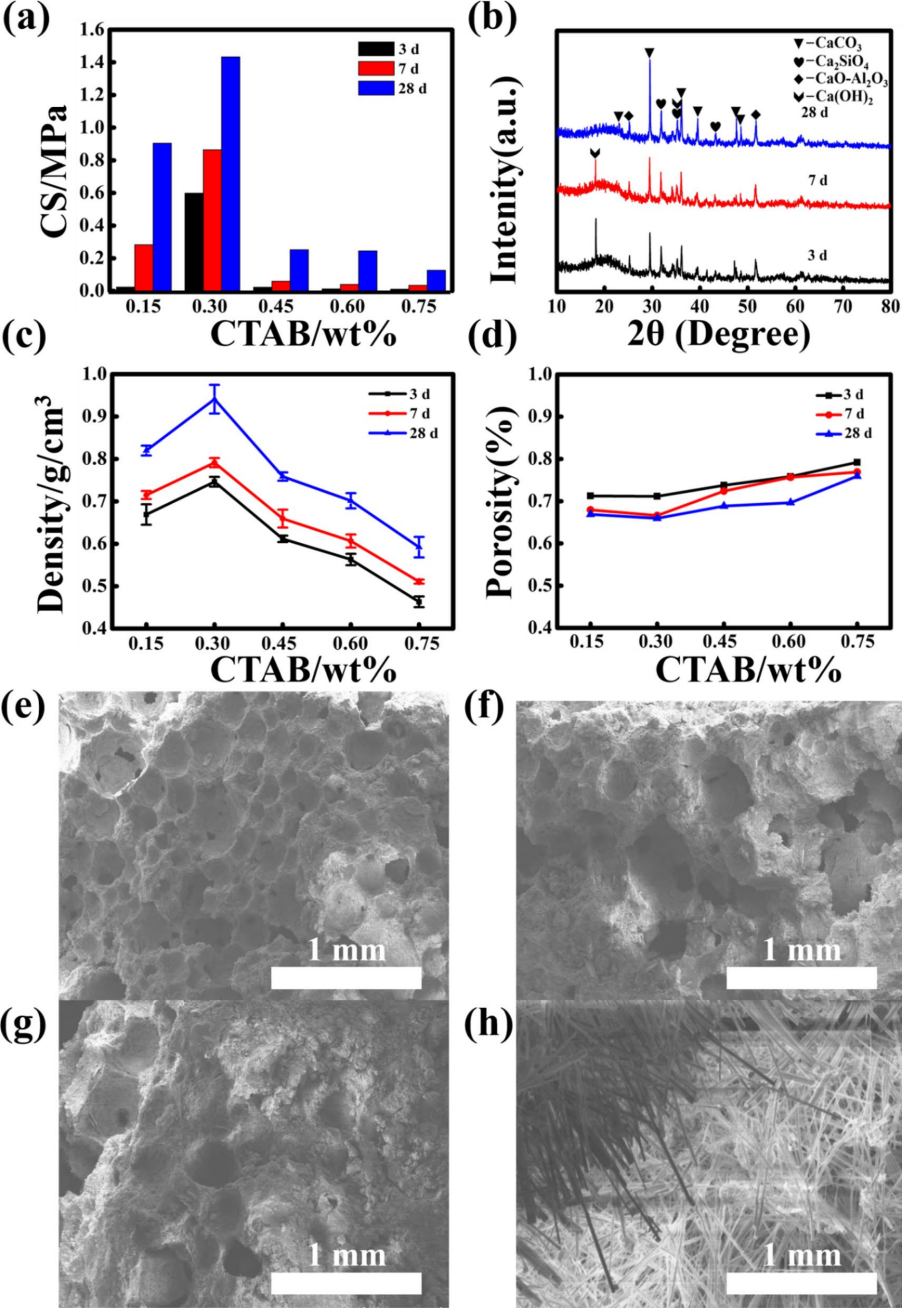
Properties and microstructure of expansive materials with different doses of CTAB. (**a**) compressive strength, (**b**) XRD pattern of C-2 cured for 3 d, 7 d, 28 d, (**c**) density, (**d**) porosity, (**e**,**f**,**g**,**h**) SEM images of C-2 cured for 3 d, 7 d, 28 d and the microgram of group C-2 cured for 28 d.

It can be known from the above experiment that 2% sodium silicate is the best addition amount for this sample. In order to further explore the influence of different amounts of CTAB on the mechanical properties of the samples, the following experiments were designed. Add 0.15%, 0.3%, 0.45%, 0.6% and 0.75% of CTAB, respectively. After curing for 3 days, 7 days, and 28 days, the compressive strength was tested. The experimental results are shown in Fig. 4a. Only the compressive strength of the sample with 0.3% CTAB addition amount reached above 1 MPa. During the foaming process, CTAB can be adsorbed on the calcium silicate contained in the smelting slag, changing its surface potential and dispersion^48,49^. This affects the formation of hydrated calcium silicate gel, which in turn affects the compressive strength of the sample. It is shown in Fig. 4b that the content of hydration products in the sample increases with the extension of curing time. A positive correlation between sample density and compressive strength was shown in Fig. 4c, which is consistent with the previous test results.

**Figure 5:**
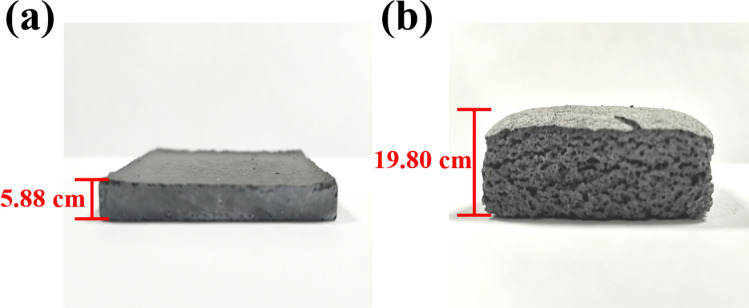
Contrast before and after foaming. (**a**) Before foaming; (**b**) After foaming.

**Figure 6:**
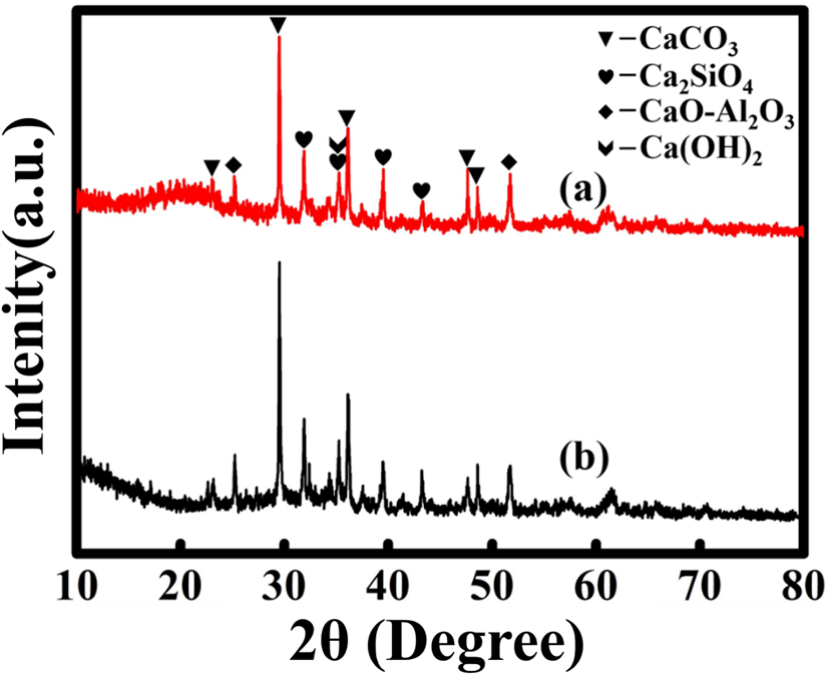
XRD pattern of the expansion material before foaming and after foaming. (**a**) After foaming; (**b**) Before foaming.

When CTAB is added above 0.45%, the surface tension of the slurry is lower, making the foaming process easier. As shown in Fig. 4d, the increase of CTAB content can increase the porosity of the sample, which directly leads to the decrease of the compressive strength of the sample. The rock compressive strength of sample C-2 after 28 days of curing is 1.43 MPa, which meets the standard requirements. Therefore, the composition ratio of sample C-2 can be used as the ratio of roof-contacted filling materials in the goaf of the mine. Figure 4e,h shows the SEM image of C-2 samples cured for 3, 7 and 28 days and the microgram of samples cured for 28 days. As can be seen from the figure, there are many needle-like cement hydration products on the surface of the sample, which ensures the strength of the sample. The porosity of the material prepared with the best ratio is as high as 65.9% (above 3 times, Fig. 5), which improves the efficiency of roof-contacted filling. At the same time, the physical phase of the material does not change with the expansion of the sample, which means that the expansion process only changes the physical properties of the material. The phase was shown in Fig. 6. At the same time, the utilization rate of copper-nickel smelting slag with the formula can reach 70%. The higher utilization rate of smelting slag can effectively reduce the environmental pollution caused by improper processing.

## Conclusions

In conclusions, an expansion material for roof-contacted filling of the goaf of a mine prepared by using copper–nickel smelting slag is prepared. The influence of sand-cement ratio, sodium silicate as well as CTAB on this material and the mechanical properties of the material itself are explored. It is found that the sand-to-cement ratio has a greater impact on the compressive strength of the sample. The addition of sodium silicate has little effect on the compressive strength and porosity of the material, nevertheless, it greatly increases the strength of the material at the initial stage of hydration. This is because the hydroxide ions generated by the hydrolysis of the sodium silicate react with the active SiO_2_ in the smelting slag and the hydrated calcium silicate gel formed provides early strength for the material. CTAB reduces the surface tension of the slurry can be reduced and the SiO_2_ component in the smelting slag can be activated by CTAB, which makes the foaming process easier and ensures the stability of the foam. It also makes an important contribution to the formation of the early strength of the material.

## Supplementary Information

Supplementary Figures.
