# Supplementary material for: A new expansion material used for roof-contacted filling based on smelting slag
Source: Sci Rep. 2021 Jan 28;11:2607. doi: 10.1038/s41598-021-81891-4 (PMC7844011; doi:10.1038/s41598-021-81891-4)
Supplement: Supplementary file 1 — Supplementary Figures. [file 41598_2021_81891_MOESM1_ESM.pdf]

A New Expansion Material Used for Roof-Contacted Filling Based on Smelting Slag

Hua Na[1], Guocheng Lv[1]*, Lijuan Wang[1], Libing Liao[1], Dan Zhang[2], Lijie Guo[2]*, Wenchen Li[2]

1. School of Materials Science and Technology, Beijing Key Laboratory of Materials Utilization of Nonmetallic Minerals and Solid Wastes, China University of Geosciences, Beijing 100083, China;

2. BGRIMM Technology Group, Beijing 100160, China;

Author to whom correspondence should be addressed:

E-Mail: guochenglv@cugb.edu.cn;

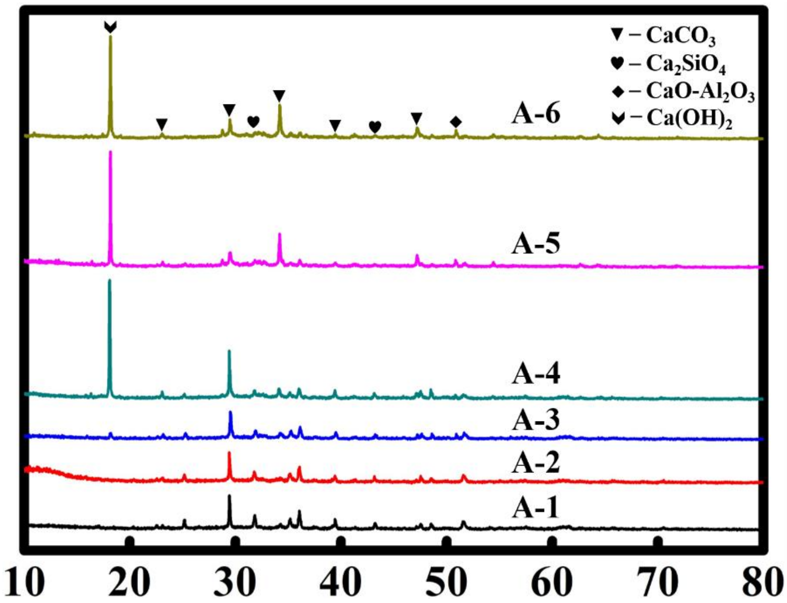

Figure S1. XRD pattern of A-1 to A-6 cured for 28 d.

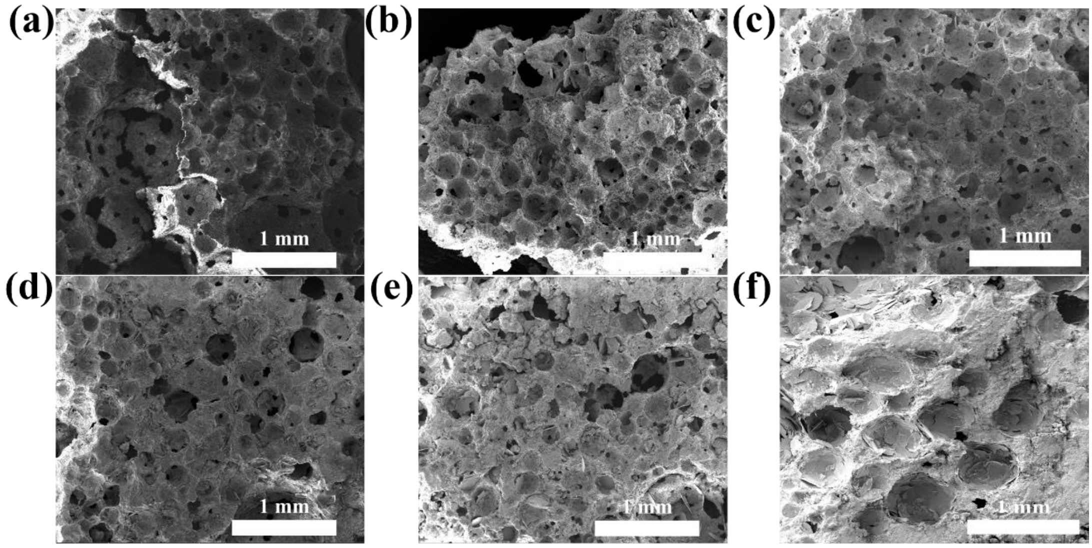

Figure S2. SEM images of A-1 to A-6 cured for 28 d.

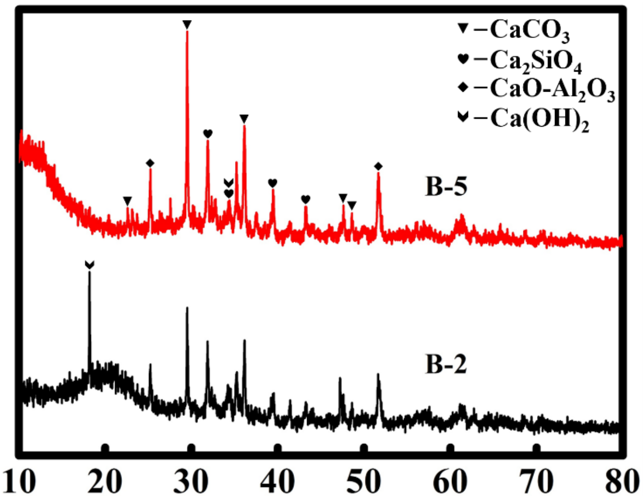

Figure S3. XRD patterns of B-2 and B-5 cured for 3 d.

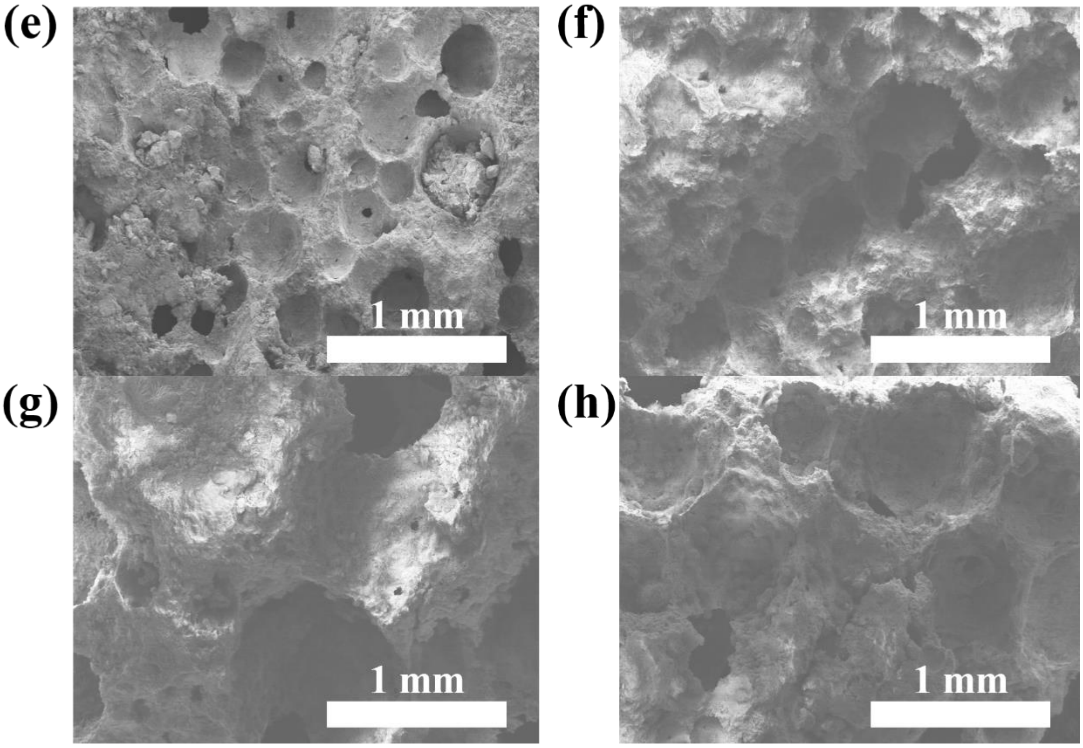

Figure S4. SEM images of B-1, B-3, B-4, B-5 cured for 28 d.
